# Supplementary material for: Metheor: Ultrafast DNA methylation heterogeneity calculation from bisulfite read alignments
Source: PLoS Comput Biol. 2023 Mar 20;19(3):e1010946. doi: 10.1371/journal.pcbi.1010946 (PMC10062925; doi:10.1371/journal.pcbi.1010946)
Supplement: S1 Text — (PDF) [file pcbi.1010946.s001.pdf]

Supplementary Material for Metheor: Ultrafast DNA methylation heterogeneity calculation from bisulfite read alignments

Dohoon Lee, Bonil Koo, Jeewon Yang and Sun Kim

March 2, 2023

1 Supplementary information

In this supplementary information, we describe both the details of the algorithms used in Metheor implementation and simulated data preparation used for benchmark.

1.1 Data structure and notations

Metheor parses bisulfite alignment files using `rust-htslib` library [1]. While iterating through the aligned reads, we utilize a compact representation of aligned bisulfite reads to keep the memory requirement as minimum as possible. Throughout the discussion, we denote an aligned bisulfite read as r, which is internally represented as a composite data structure consisting of the following member variables:

- `start_pos`: The (0-based) leftmost position with regard to the reference genome where the read was aligned. Its value is accessed by a method GETSTARTPOS.
- `end_pos`: The (0-based) rightmost position with regard to the reference genome where the read was aligned. Its value is accessed by a method GETENDPOS.
- `cpgs`: A vector of CpGs covered by the read. The number of CpGs is accessed by a method GETNUMCPGS, and the position of the first (leftmost) CpG is accessed by a method GETFIRSTCPGPOSITION.

Individual CpG on the sequencing read is also represented by a dedicated data structure with the following member variables:

- `abspos`: The (0-based) absolute position of the CpG with regard to the reference genome (i.e., corresponding chromosome). Its value is accessed by a method GETPOSITION.
- `relpos`: The (0-based) relative position of CpG within the sequencing read. Maintaining this variable facilitates simple operations for CpGs within a read.

- `is_methylated`: A boolean value denoting whether the CpG is determined to be methylated in the read. Its value is accessed by a method isMethylated.

The methylation state of each CpG can be easily determined by directly parsing the Bismark methylation string (BAM tag specified as 'XM') [2]. Therefore, it is required for an input BAM file to have Bismark methylation strings in order to run Metheor. For those who prefer methylation-aware aligners other than Bismark, we provide an auxiliary command named `tag` to add XM-tag automatically to any BAM file.

In the following sections, we discuss the biological motivations of each methylation heterogeneity measure and algorithmic details for the computation of them in Metheor.

1.2 Computation of proportion of discordant reads (PDR)

Due to the processive nature of the catalysis driven by DNA methyltransferases and demethylases, local DNA methylation states are correlated with each other. In other words, it is highly likely to observe a methylated CpG when the nearby CpGs are methylated, and vice versa. In cancers, this local homogeneity of DNA methylation states is known to be eroded and is also known to be associated with the clinical outcome of a patient. In this regard, PDR measures how much the local homogeneity of DNA methylation states is eroded in a given genomic region (S1 Fig). In the definition of PDR [3], a read is defined as *discordant* if CpGs covered by the read has both methylated and unmethylated states (representing *locally disordered* methylation states), otherwise a read is defined as *concordant*. PDR is a CpG-wise measure, that is, a single PDR value is assigned for a CpG. For a set of reads covering the CpG, PDR is defined as a proportion of discordant reads. For example, a CpG covered by reads harboring consistent methylation states (either fully-methylated or fully-unmethylated) has PDR of 0, and otherwise has PDR greater than 0.

In this section, we describe how the computation of PDR is implemented in Metheor using a **single sweep** of the given alignment file. As a result, a hashmap associating each CpG to a corresponding PDR value is obtained by Algorithm 1. While iterating bisulfite reads in the given alignment file, we maintain a temporary hashmap T linking each CpG to a number of associated concordant/discordant reads or n_c and n_d. For each read r and for each CpG within the read, n_c and n_d in T is continuously updated based on the concordance state of read r, which is determined by isDiscordant method (Algorithm 2). Note that by assuming that the alignment file is sorted by genomic coordinates in an increasing order, it can be ensured that a CpG will not be processed any more when we encounter a read whose leftmost CpG position is greater than that CpG. By finalizing those CpGs to the hashmap H, we can reduce the amount of working set in T.

Algorithm 1 Computation of proportion of discordant reads (PDR)

Input: $\mathbf{R}$, a set of aligned bisulfite reads
Input: d, minimum read depth
Input: p, minimum number of CpGs in a read
Input: q, minimum mapping quality
Input: $\mathbf{C}$, (optional) target CpG set
Output: a hashmap $H : CpG \to f_{pdr}$ ▷ Map CpG to PDR

```
Initialize an empty hashmap T : CpG → (n_c, n_d)
                        ▷ Map CpG to (#concordant reads, #discordant reads)
Initialize an empty hashmap H : CpG → f_pdr
for r in R do
    if C is specified then r ← filterCpgs(r, C) end if
    if getNumCpgs(r) < p then continue end if
    if getMAPQ(r) < q then continue end if
    x ← getFirstCpgPosition(r)
    for cpg, (n_c, n_d) in T do
        if getPosition(cpg) < x then
            if n_c + n_d < d then continue end if
            f_pdr ← n_d/(n_c + n_d)
            H[cpg] ← f_pdr
            Remove cpg from T
        end if
    end for
    d ← isDiscordant(r)
    for cpg in getCpgs(r) do
        if d = True then
            incrementDiscordant(T[cpg])
        else
            incrementConcordant(T[cpg])
        end if
    end for
end for
for cpg, (n_c, n_d) in T do
    if n_c + n_d < d then continue end if
    f_pdr ← n_d/(n_c + n_d)
    H[cpg] ← f_pdr
end for
```

Algorithm 2 Determining whether a bisulfite read is discordant

```
procedure isDiscordant(r)
    C ← getCpgs(r)
    s ← isMethylated(C[0])                    ▷ Get methylation state of the first CpG
    for cpg in getCpgs(r) do
        if isMethylated(cpg) ≠ s then
            return True
        end if
    end for
    return False
end procedure
```

1.3 Computation of local pairwise methylation discordance (LPMD)

In this study, we introduce LPMD as a new measure to quantify the local concordance of DNA methylation states. The conceptual basis of LPMD is similar to PDR as they both are aware of local homogeneity of DNA methylation states, but they are different in that LPMD explicitly takes the distance between CpGs into consideration (S2 Fig). In detail, LPMD is defined as a fraction of CpG pairs within a given range of genomic distance (i.e., CpG pairs more distant than m bp and closer than M bp) and therefore, LPMD is defined for a *pair* of CpGs, but not for a single CpG. Importantly, we note that LPMD does not depend on length of sequencing read. Considering that there is an increased tendency of observing discordant reads solely by chance based on definition in PDR, LPMD proves to be a novel method for measuring DNA methylation heterogeneity.

The goal of Algorithm 3 is to obtain a hashmap associating a pair of CpG to a corresponding LPMD value. This algorithm also computes LPMD value for each pair of CpGs after a **single sweep** of the given alignment file. Extracting all pairwise concordance/discordance statistics is performed using computePairwiseStats method (Algorithm 4), which is a variation of two-pointer algorithm that keeps the minimal set of CpGs needed for further pairwise examinations.

Algorithm 3 Computation of local pairwise methylation discordance (LPMD)

Input: $\mathbf{R}$, a set of aligned bisulfite reads
Input: q, minimum mapping quality
Input: m, minimum distance between CpG pairs
Input: M, maximum distance between CpG pairs
Input: $\mathbf{C}$, (optional) target CpG set
Output: a hashmap $H : (CpG_1, CpG_2) \rightarrow f_{lpmd}$ ▷ Map CpG pair to LPMD

Initialize an empty hashmap $T : (CpG_1, CpG_2) \rightarrow (n_c, n_d)$
▷ Map CpG pair to (#concordant reads, #discordant reads)
▷ n_c and n_d are initialized to 0

for r in $\mathbf{R}$ **do**
    if $\mathbf{C}$ is specified **then** $r \leftarrow$ FILTERCPGS$(r, \mathbf{C})$ **end if**
    if GETMAPQ$(r) < q$ **then** continue **end if**
    $n_c, n_d, S \leftarrow$ COMPUTEPAIRWISESTATS(r, m, M)
    ▷ S: Vector of all pairwise concordances (CpG_1, CpG_2, s: concordance state)
    for (cpg_1, cpg_2, s) in S **do**
        if s = discordant **then**
            INCREMENTDISCORDANT$(T[(cpg_1, cpg_2)])$
        else
            INCREMENTCONCORDANT$(T[(cpg_1, cpg_2)])$
        end if
    end for
end for
Initialize an empty hashmap $H : (CpG_1, CpG_2) \rightarrow f_{lpmd}$
for $(cpg_1, cpg_2), (n_c, n_d)$ in T **do**
    $f_{lpmd} \leftarrow n_d/(n_c + n_d)$
    $H[(cpg_1, cpg_2)] \leftarrow f_{lpmd}$
end for

Algorithm 4 Extracting all pairwise concordance/discordance statistics from a read

```
procedure computePairwiseStats(r, m, M)
    n_c, n_d ← 0
    a ← −1                                              ▷ Pointer for the leftmost anchor
    Initialize a vector of anchor-CpGs A
    Initialize a vector of CpG pair statistics S: (CpG_1, CpG_2, s)
    for cpg in getCpGs(r) do
        if a ≠ −1 then
            while computeDistance(cpg, a) > M and A is not empty do
                remove first element in A
                if A is not empty then
                    a ← position of the first element in A
                else
                    a ← −1
                end if
            end while
        end if                                          ▷ Remove irrelevant anchor-CpGs from A
        for a in A do
            if computeDistance(cpg, a) < m then continue end if
            if cpg and a has the same methylation state then
                n_c ← n_c + 1 and push (a, cpg, concordant) to S
                                                        ▷ Increment #concordant reads by 1
            else
                n_d ← n_d + 1 and push (a, cpg, discordant) to S
                                                        ▷ Increment #discordant reads by 1
            end if
        end for
        if a = −1 then a ← position of cpg
        push cpg to A
    end for
    return (n_c, n_d, S)
end procedure
```

1.4 Computation of methylation haplotype load (MHL)

The concept of MHL is also based on the local homogeneity of DNA methylation states, or co-methylation, due to the processivity of enzymes responsible for methylation and demethylation of cytosines [4]. While PDR and LPMD focus on how much the tendency of co-methylation is perturbed in the given population of cells, MHL focuses on how well the methylation haplotypes (i.e., *stretch* of consecutively methylated CpGs) are conserved throughout the cell population for a given genomic region (S3 Fig). The basic concept of MHL is to systematically identify the genomic blocks harboring CpGs with tightly coupled methylation states. In detail, MHL is computed as a fraction of observed *fully methylated stretches* out of the all stretches of every possible lengths. Notably, the authors proposed that giving more weights to longer methylated stretches heuristically worked well. Altogether, an intuitive definition of MHL can be formulated as below.

$$\mathrm{MHL} = \frac{\sum_{l=1}^{L} w_l \times p(\mathrm{x}_m^l)}{\sum_{l=1}^{L} w_l}, \tag{1}$$

where $p(\mathrm{x}_m^l)$ denotes the fraction of fully methylated stretches of length l out of all possible stretches, and w_l represents the weight given to a fraction of methylated stretch of length l. In the implementation of Metheor, we sticked to $w_l = l$ according to the suggestion of the original authors [4].

In the current version of Metheor implementation, a hashmap associating each CpG to a corresponding MHL value is obtained by Algorithm 5 with a **single sweep** of read alignments. For an efficient computation of MHL, we maintain a composite data structure named `AssociatedReads` that contains the summary statistics of methylated stretches associated with each CpG. `AssociatedReads` consists of the following member variables:

- `pos`: Position of CpG of interest for which this object summarizes the information of associated reads.
- `stretch_info`: Hashmap $S : l \rightarrow n_l$, which represents the count n_l of methylated stretch of length l.
- `num_cpgs`: Vector of CpG counts for all the associated reads. Accessed by a method GETNUMCPGS.
- `max_num_cpgs`: max(`num_cpgs`). Accessed by a method GETMAXNUMCPGS.

In particular, introducing a hashmap `stretch_info` is the key idea for the efficient computation of MHL. Using methylation states of CpGs covered by a read, we can compute a read-wise statistics of methylated stretch as Algorithm 6, and using `stretch_info`, we can easily compute MHL values using Algorithm 7.

Algorithm 5 Computation of methylation haplotype load (MHL)

```
Input: R, a set of aligned bisulfite reads
Input: d, minimum read depth
Input: p, minimum number of CpGs in a read
Input: q, minimum mapping quality
Input: C, (optional) target CpG set
Output: a hashmap H : CpG → f_mhl                ▷ Associates each CpG with MHL
Initialize an empty hashmap T : CpG → AssociatedReads
                                   ▷ Associates each CpG with summary statistics
Initialize an empty hashmap H : CpG → f_mhl
for r in R do
    if C is specified then r ← filterCpgs(r, C) end if
    if getNumCpgs(r) < p then continue end if
    if getMAPQ(r) < q then continue end if
    x ← getFirstCpgPosition(r)
    for cpg, a in T do                           ▷ a : AssociatedReads
        if getPosition(cpg) < x then
            if n_c + n_d < d then continue end if
            f_mhl ← computeMhl(a)
            H[cpg] ← f_mhl
            Remove cpg from T
        end if
    end for
    for cpg in getCpgs(r) do
        a ← T[cpg]
        addNumCpGs(a, getNumCpGs(r))
        addStretchInfo(a, getStretchInfo(r))
    end for
end for
for cpg, a in T do                               ▷ a : AssociatedReads
    if n_c + n_d < d then continue end if
    f_mhl ← computeMhl(a)
    H[cpg] ← f_mhl
    Remove cpg from T
end for
```

Algorithm 6 Extracting metylation stretch information from a read

procedure getStretchInfo(r)
    Initialize an empty hashmap $S : l \rightarrow n_l$
    $l_{curr} \leftarrow 0$
    for cpg in getCpgs(r) **do**
        if isMethylated(cpg) **then**
            $l_{curr} \leftarrow l_{curr} + 1$
            for l in 1 to l_{curr} **do**
                $S[l] \leftarrow S[l] + 1$
            end for
        else
            $l_{curr} \leftarrow 0$
        end if
    end for
    return S
end procedure

Algorithm 7 Computing MHL from summary statistics of associated reads

procedure computeMhl(a: `AssociatedReads`)
    $f_{mhl} \leftarrow 0$
    $l_{sum} \leftarrow 0$
    for l in 1 to getMaxNumCpgs(a) **do**
        $l_{sum} \leftarrow l_{sum} + l$
    end for
    for l, n_l in getStretchInfo(a) **do**
        $d \leftarrow 0$
        for n_c in getNumCpGs(a) **do**
            $d \leftarrow d + (n_c - l + 1)$
        end for
        $f_{mhl} \leftarrow f_{mhl} + (l \times n_l)/d$
    end for
    $f_{mhl} \leftarrow f_{mhl}/l_{sum}$
    return f_{mhl}
end procedure

1.5 Computation of epipolymorphism (PM) and methylation entropy (ME)

PM [5] and ME [6] are closely related measures that are defined for phased methylation states, or epialleles, so we discuss these two measures together in this section (S4 Fig). The goal of these measures is to quantify the diversity of DNA methylation states of a given cell population. However, identifying the whole phased DNA methylation states of each cell to compute and calculating their diversity is not feasible. To circumvent this problem practically, PM and ME measure the diversity of the *methylation patterns*, or epialleles, formed by four consecutive CpGs covered by a single. bisulfite read. There are $2^4 = 16$ possible methylation patterns in total. Note that the concordance/discordance of the methylation states of nearby CpGs are not of interest to PM and ME, but they are only interested in how diverse those 16 methylation patterns are. To distinguish the four consecutive CpGs themselves from the methylation states of them, we term the former as *CpG quartet* and the latter as *methylation patterns* or *epialleles*.

The following algorithms (Algorithm 8 and 9) show how we obtain a hashmap H associating each CpG quartet with corresponding PM and ME values through a **single sweep** of the alignment file.

Algorithm 8 Computation of epipolymorphism (PM)

Input: $\mathbf{R}$, a set of aligned bisulfite reads
Input: d, minimum read depth
Input: q, minimum mapping quality
Input: $\mathbf{C}$, (optional) target CpG set
Output: A hashmap $H : Q \rightarrow f_{pm}$
▷ Map quartet Q to PM, where Q: $(CpG_1, CpG_2, CpG_3, CpG_4)$

Initialize an empty hashmap $T : Q \rightarrow \underbrace{[0, ..., 0]}_{16}$
▷ Map quartet Q to epiallele counts
for r in $\mathbf{R}$ **do**
    if $\mathbf{C}$ is specified **then** $r \leftarrow$ filterCpgs($r, \mathbf{C}$) **end if**
    if getMAPQ(r) $< q$ **then** continue **end if**
    for ($quartet, pat$) in getQuartetsAndPatterns(r) **do**
        increment T[$quartet$][pat] by 1
        ▷ pat: Integer representation of epialleles. e.g., 0=0000, 11=1011, 15=1111
    end for
end for
Initialize an empty hashmap $H : Q \rightarrow f_{pm}$
for ($quartet, counts$) in T **do**
    $s \leftarrow sum(counts)$
    if $s < d$ **then** continue **end if**
    $f_{pm} \leftarrow 1 - \sum_{c \in counts} (\frac{c}{s})^2$
    $H[quartet] \leftarrow f_{pm}$
end for

Algorithm 9 Computation of methylation entropy (ME)

Input: $\mathbf{R}$, a set of aligned bisulfite reads
Input: d, minimum read depth
Input: q, minimum mapping quality
Input: $\mathbf{C}$, (optional) target CpG set
Output: A hashmap $H : Q \rightarrow f_{me}$
▷ Map quartet Q to ME, where Q: $(CpG_1, CpG_2, CpG_3, CpG_4)$

Initialize an empty hashmap $T : Q \rightarrow \underbrace{[0, ..., 0]}_{16}$
▷ Map quartet Q to epiallele counts
for r in $\mathbf{R}$ **do**
  if $\mathbf{C}$ is specified **then** $r \leftarrow$ filterCpgs$(r, \mathbf{C})$ **end if**
  if getMAPQ$(r) < q$ **then** continue **end if**
  for $(quartet, pat)$ in getQuartetsAndPatterns(r) **do**
    increment T$[quartet][pat]$ by 1
      ▷ pat: Integer representation of epialleles. e.g., 0=0000, 11=1011, 15=1111
  end for
end for
Initialize an empty hashmap $H : Q \rightarrow f_{me}$
for $(quartet, counts)$ in T **do**
  $s \leftarrow sum(counts)$
  if $s < d$ **then** continue **end if**
  $f_{me} \leftarrow -0.25 \times \sum_{c \in counts} \frac{c}{s}(\log_2 \frac{c}{s})$
  $H[quartet] \leftarrow f_{me}$
end for

1.6 Computation of fraction of discordant read pairs (FDRP) and quantitative FDRP (qFDRP)

FDRP and qFDRP are measures of epigenetic diversity within a cell population that were first proposed in [7]. While PM and ME quantify the epiallelic diversity in terms of CpG quartets, FDRP and qFDRP allow the computation of epiallelic diversity in a single CpG resolution. The key principle underlying the FDRP and qFDRP is as follows. When epialleles are perfectly homogeneous for a short genomic region, any two sequencing reads aligned to that region will have identical methylation states for CpGs that are common to the two reads. On the other hand, as epialleles become more diverse, it is more likely to observe a read pair that have different methylation states for common CpGs. Based on this notion, FDRP and qFDRP compute a CpG-wise epigenetic diversity by examining pairs of sequencing reads covering the CpG. Since the time required for all pairwise examination of sequencing reads increases exponentially along with the sequencing depth, the authors adopt a read sampling strategy to make those measures be computed in a feasible time. Therefore, the maximum number of sampled reads, or M, is a crucial parameter modulating the balance between the precision of the measures and the computing time.

Algorithm 10 and 11 show how Metheor obtains a hashmap associating each CpG with FDRP and qFDRP values. Like all the other measures, Metheor computes FDRP and qFDRP using a **single sweep** of sequencing reads. Similarly to MHL, FDRP and qFDRP require their own `AssociatedReads` data structure tailored for the efficient calculation of the measures.

- `pos`: Position of CpG of interest for which this object summarizes the information of associated reads.
- `reads`: Vector of sequencing reads. Maximum length of this vector is kept to `max_depth` by reservoir sampling.
- `num_total_read`: Number of the reads actually covering the CpG of interest.
- `num_sampled_read`: Number of reads in the current set of sampled reads.
- `max_depth`: Maximum number of reads allowed for sampled reads.

To reduce the memory usage during iteration and perform read sampling efficiently, Metheor utilizes a reservoir sampling when the number of reads covering the CpG exceeds `max_depth` or parameter D. For convenience, we will denote `num_sampled_read` as n in this section. When the CpG is ready to be finalized (i.e., it is guaranteed that no more reads cover the CpG), we examine all the $n(n-1)/2$ read pairs and determine whether the pair is discordant (i.e., at least one CpG common to the two reads have different methylation state) or concordant (i.e., all CpGs common to the two reads have identical methylation states) to compute FDRP. In the case of qFDRP, the normalized hamming distance (i.e., the number of CpGs with different methylation states divided by the number of common CpGs) is used instead. Finally, we obtain FDRP by dividing the number of discordant read pairs by $n(n-1)/2$, and we obtain qFDRP by dividing the sum of normalized hamming distance also by $n(n-1)/2$.

Algorithm 10 Computation of fraction of discordant read pairs (FDRP)

Input: $\mathbf{R}$, a set of aligned bisulfite reads
Input: d, minimum read depth
Input: D, maximum read depth to consider (i.e., reads will be sampled up to D reads)
Input: l, minimum number of overlapped bases to say the two reads overlap
Input: q, minimum mapping quality
Input: $\mathbf{C}$, (optional) target CpG set
Output: A hashmap $H : CpG \rightarrow f_{fdrp}$ ▷ Map CpG with FDRP

```
Initialize an empty hashmap T : CpG → AssociatedReads

Initialize an empty hashmap H : CpG → f_fdrp
                                                ▷ Map CpG to the sampled reads
for r in R do
    if C is specified then r ← filterCpgs(r, C) end if
    if getMAPQ(r) < q then continue end if
    x ← getFirstCpgPosition(r)
    for (cpg, a) in T do                        ▷ a : AssociatedReads
        if getPosition(cpg) < x then
            if getNumTotalRead(a) < d then continue end if
            f_fdrp ← computeFdrp(a)
            H[cpg] ← f_fdrp
            Remove cpg from T
        end if
    end for
    for cpg in getCpgs(r) do
        a ← T[cpg]
        addRead(a, r)                           ▷ Read sampling occurs here (if needed)
    end for
end for
for (cpg, a) in T do                            ▷ a : AssociatedReads
    if getNumTotalRead(a) < d then continue end if
    f_fdrp ← computeFdrp(a)
    H[cpg] ← f_fdrp
    Remove cpg from T
end for
```

Algorithm 11 Computation of quantitative FDRP (qFDRP)

```
Input: R, a set of aligned bisulfite reads
Input: d, minimum read depth
Input: D, maximum read depth to consider (i.e., reads will be sampled up to D reads)
Input: l, minimum number of overlapped bases to say the two reads overlap
Input: q, minimum mapping quality
Input: C, (optional) target CpG set
Output: A hashmap H : CpG → f_qfdrp                              ▷ Map CpG with FDRP
Initialize an empty hashmap T : CpG → AssociatedReads

Initialize an empty hashmap H : CpG → f_qfdrp
                                                       ▷ Map CpG to the sampled reads
for r in R do
    if C is specified then r ← filterCpgs(r, C) end if
    if getMAPQ(r) < q then continue end if
    x ← getFirstCpgPosition(r)
    for (cpg, a) in T do                                   ▷ a : AssociatedReads
        if getPosition(cpg) < x then
            if getNumTotalRead(a) < d then continue end if
            f_qfdrp ← computeQfdrp(a)
            H[cpg] ← f_qfdrp
            Remove cpg from T
        end if
    end for
    for cpg in getCpgs(r) do
        a ← T[cpg]
        addRead(a, r)                          ▷ Read sampling occurs here (if needed)
    end for
end for
for (cpg, a) in T do                                       ▷ a : AssociatedReads
    if getNumTotalRead(a) < d then continue end if
    f_qfdrp ← computeQfdrp(a)
    H[cpg] ← f_qfdrp
    Remove cpg from T
end for
```

1.7 Insights into the algorithmic advantages of Metheor

The main algorithmic advantage of Metheor comes from the fact that it only reads through the entire BAM file only once (Figure 1C in the main text). In this section, we call it as *read-centric* approach, as each sequencing read is processed only once. On the other hand, existing methods for the computation of PDR, FDRP, qFDRP (by WSHPackage) and MHL (by the Perl script provided by the authors) takes *CpG-centric* approach that iterates through each CpG specified by a user, and fetches the reads covering that CpG using BAM index (Figure 1D in the main text). Even though the alignment index allows the access of reads quickly, such read access consumes the largest portion of the running time in the execution of the program. In this context, we here provide a brief insight into the algorithmic advantages in Metheor along with our empirical observations supporting the discussion.

The overhead of read-centric approach is that reads with no CpGs are also accessed. On the other hand, the overhead of CpG-centric approach is that redundant read accesses are needed for reads with more than one CpGs. To numerically compare the effect of the two potential drawbacks, we first denote the total number of aligned reads as n. Then, the number of read accesses in read-centric approach is trivially guaranteed to be n. For CpG-centric approach, we can notice that the number of access for a read is exactly same to the number of CpGs covered by the read. Therefore, the total number of read access in CpG-centric approach is the total number of individual cytosines covered by sequencing reads. When we denote the average number of covered CpGs per read as λ, we can conclude that CpG-centric approach requires $\lambda \times n$ read accesses. Thus, the value of λ for a dataset decides which approach is favored over the other.

To assess the empirical value of λ in general RRBS experiments, we collected the statistics from a large number of 928 public RRBS experiments on CCLE cell lines [8]. Raw RRBS sequencing reads were downloaded from SRA under study accession SRP186687, adapter-trimmed using trim-galore, and aligned to the reference genome with Bismark. As shown in Figure 1E in the main text, we discovered that the values of λ is greater than 1 in every case, supporting that read-centric approach in realistic samples.

1.8 Benchmarking the running time of Metheor for only a subset of CpGs

As described in the main text, the theoretical running time of Metheor may not be better than CpG-centric benchmark implementations if only a subset of CpGs are considered for the calculation of DNA methylation heterogeneity. To examine whether Metheor still show better empirical performance even if fewer CpGs are considered, we conducted a benchmark experiment by subsampling the set of target CpGs. We subsampled 5%, 10%, 20%, 30%, 40%, 50%, 60%, 70%, 80%, 90% and 100% of CpGs and let metheor and WSHPackage compute PDR, FDRP and qFDRP only for those CpGs. As a result, we observed that Metheor ran faster than WSHPackage even when 5% of CpGs were considered (S5 Fig).

1.9 Robustness of LPMD against the choice of genomic distance window

To examine whether the trends of LPMD values are robust against the choice of genomic distance window, we computed Spearman's correlation between two LPMD values with different distance window setting. As a result, we could show that LPMD values computed with 2∼4bp window and 2∼28bp window had high correlation (∼0.96, S6 Fig), suggesting that the choice of distance window is not critical for LPMD analyses as long as the same window is used for all samples subjected to the comparison of LPMD values.

1.10 RRBS read simulation

To benchmark the performance of Metheor, we simulated a realistic RRBS data with various numbers of reads according the the procedure described in this section. First, the whole hg38 reference genome was digested *in silico* by a virtual restriction enzyme MspI having restriction site 5'-C|CGG-3'. It produced 2,317,722 genomic fragments in total. To imitate the experimental size-selection procedure, we only kept the fragments with size range between 50-200bp, and obtained 531,112 fragments. Then, we generated a simulated RRBS dataset by sampling reads from those fragments. Nine different sequencing data were generated with the number of sampled reads configured as follows: (1) 500K, (2) 2M, (3) 4M, (4) 8M, (5) 16M, (6) 20M, (7) 50M, (8) 100M and (9) 200M. To simulate a sequencing read, we randomly sampled a restriction fragment with replacement and determined the methylation level β using distribution $Beta(0.25, 0.25)$. For each CpG covered by the read, it was methylated with probability β, i.e., the cytosine is converted to thymine with probability $1 - \beta$. At the same time, a Bismark XM-tag was also simulated for each read accordingly. The orientation of the read (forward/reverse) was randomly determined with probability of 0.5. For convenience, read mapping quality was fixed to 40, base quality was fixed to 41 ('J' in ASCII representation) and no sequencing errors were introduced. Finally, all the simulated aligned reads were written to BAM files.

1.11 Pseudo-WGBS read simulation

To simulate pseudo-WGBS reads (S7 Fig), we followed a read simulation strategy used in [7]. We especially adopted a scenario imitating DNA methylation erosion in the short genomic stretches throughout the whole genome. The whole simulation procedure is described as follows. First, we selected N random genomic regions across the genome (except chromosome 22, X and Y) in order to reduce the computational load while keeping the sequenced region unbiased for the whole genome. Note that this is why we call this simulated dataset pseudo-WGBS, not WGBS. All regions had fixed size of 50kbp, and no regions were allowed to have bases other than A, C, G and T. Then, for each region, we randomly divided the region into three segments. Each segment was forced to be at least 150bp long. We subsequently sampled sequencing reads from first and last segments (which we call first and second subregion, respectively), and kept all the CpGs on the sampled reads fully methylated. In this process, the number of sequencing reads to be sampled from those two subregions was determined proportionally to length of each corresponding region. Specifically, this number was calcu-

lated as 50kbp $\times$ (length fraction of each subregion). On the other hand, the segment in the middle (which we call erosion subregion) was processed in more sophisticated way to simulate the process of DNA methylation erosion. First of all, to represent stochastic methylation erosion occurring during cell proliferation, we sampled a random number (`replicate_num`) in the range of [2,10], following [7]. Since we should consider both the `replicate_num` and length fraction of erosion region in deciding total number of sequencing reads to be sampled from this subregion, we initially sampled small number of samples from erosion subregion and concatenated resulting FASTQ files for `replicate_num` times. Also, we added two random factors in sampling sequencing reads from erosion subregion. The first was deciding whether to erode the methylation states of CpGs within erosion subregion, and the second was to determine the level of methylation erosion. For the first factor, we randomly sampled a floating-point number in the range of [0,1] and eroded the erosion region if this random number was above 0.5, which represents erosion probability of 0.5. For the second factor, we randomly sampled an integer in the range of [1,100] and set this as level of methylation erosion using Sherman (https://www.bioinformatics.babraham.ac.uk/projects/sherman). After independently processing N regions as explained above, we concatenated all the resulting FASTQ files into a single FASTQ file to obtain the final simulated sequencing reads. This merged FASTQ file was processed with Bismark, and the resulting BAM file was used as simulated pseudo-WGBS alignment, and the simulation was done with varying N to generate FASTQ files with 1M, 2.5M, 10M and 20M sequencing reads.

1.12 Ewing sarcoma RRBS read subsampling

Finally, we downloaded a real-world Ewing sarcoma RRBS data under SRA run accession SRR5222549 as a benchmark data. The library was constructed from a ewing sarcoma tumor sample and consisted of 18.9M single-end bisulfite reads. To measure the performance of Metheor and the other tools for various sizes of sequencing data, we resampled the sequencing reads to generate differently sized raw sequencing data. As a result, five different raw sequencing data (in FASTQ format) with (1) 500K, (2) 2M, (3) 4M, (4) 8M and (5) 16M reads were generated. Sampled sequencing reads were subsequently adapter-trimmed with trim-galore!, aligned to hg38 reference genome with Bismark, and the resulting alignment files were used for benchmark.

References

1. Köster J. Rust-bio: a fast and safe bioinformatics library. Bioinformatics. 2016;32(3):444-446.

2. Krueger F, Andrews SR. Bismark: a flexible aligner and methylation caller for bisulfite-seq applications. Bioinformatics. 2011;27(11):1571–1572.

3. Landau DA, Clement K, Ziller MJ, Boyle P, Fan J, Gu H, et al. Locally disordered methylation forms the basis of intratumor methylome variation in chronic lymphocytic leukemia. Cancer cell. 2014;26(6):813–825.

4. Guo S, Diep D, Plongthongkum N, Fung HL, Zhang K, Zhang K. Identification of methylation haplotype blocks aids in deconvolution of heterogeneous tissue samples and tumor tissue-of-origin mapping from plasma DNA. Nature genetics. 2017;49(4):635–642.

5. Landan G, Cohen NM, Mukamel Z, Bar A, Molchadsky A, Brosh R, et al. Epigenetic polymorphism and the stochastic formation of differentially methylated regions in normal and cancerous tissues. Nature genetics. 2012;44(11):1207-1214.

6. Xie H, Wang M, De Andrade A, Bonaldo MDF, Galat V, Arndt K, et al. Genome-wide quantitative assessment of variation in DNA methylation patterns. Nucleic acids research. 2011;39(10):4099–4108.

7. Scherer M, Nebel A, Franke A, Walter J, Lengauer T, Bock C, et al. Quantitative comparison of within-sample heterogeneity scores for DNA methylation data. Nucleic acids research. 2020;48(8):e46–e46.

8. Ghandi M, Huang FW, Jané-Valbuena J, Kryukov GV, Lo CC, McDonald ER, et al. Next-generation characterization of the cancer cell line encyclopedia. Nature. 2019;569(7757):503–508.
