# Supplementary figures and images for: Metheor: Ultrafast DNA methylation heterogeneity calculation from bisulfite read alignments
Source: PLoS Comput Biol. 2023 Mar 20;19(3):e1010946. doi: 10.1371/journal.pcbi.1010946 (PMC10062925; doi:10.1371/journal.pcbi.1010946)

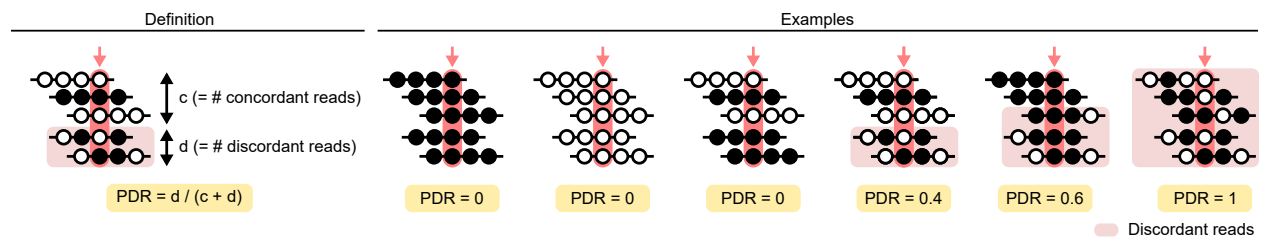

**S1 Fig.** Schematic illustration of proportion of discordant reads (PDR).

Supplement: S1 Fig — (PDF) [file pcbi.1010946.s002.pdf]

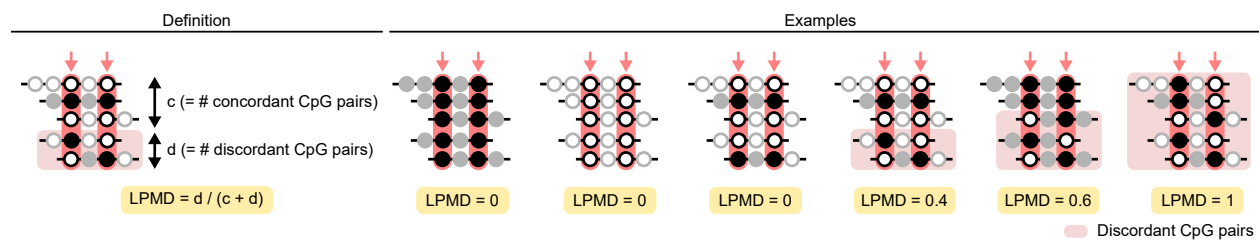

**S2 Fig.** Schematic illustration of local pairwise methylation discordance (LPMD).

Supplement: S2 Fig — (PDF) [file pcbi.1010946.s003.pdf]

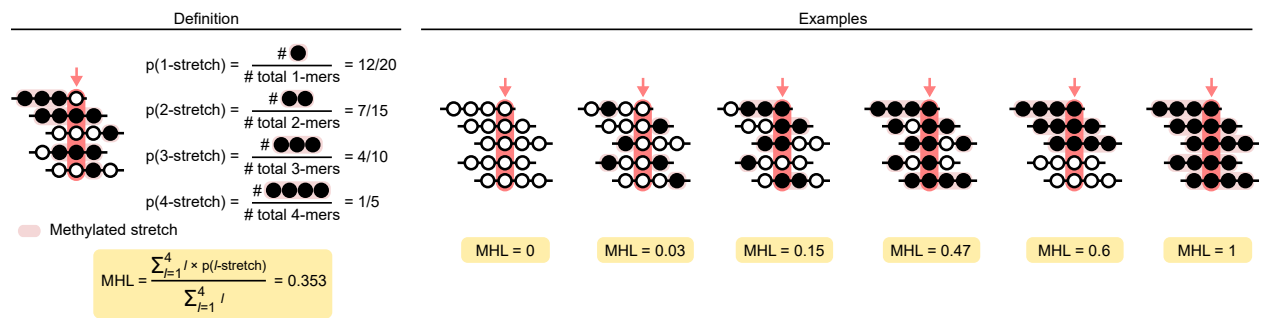

**S3 Fig.** Schematic illustration of methylation haplotype load (MHL).

Supplement: S3 Fig — (PDF) [file pcbi.1010946.s004.pdf]

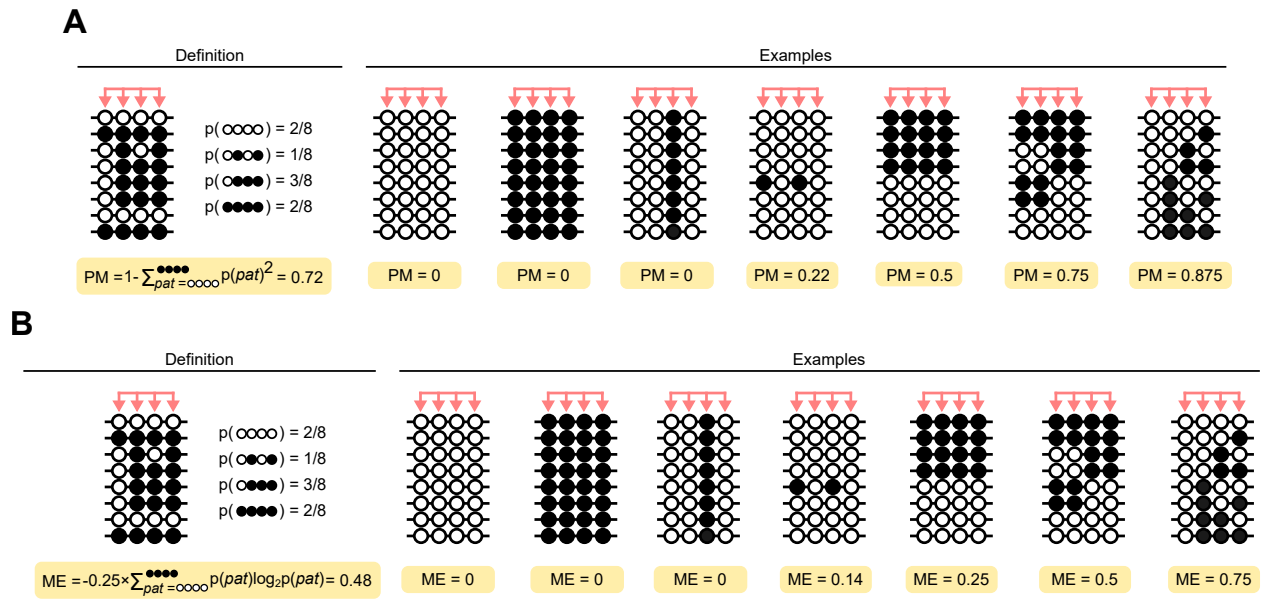

**S4 Fig.** Schematic illustration of (A) epipolymorphism (PM) and (B) methylation entropy (ME).

Supplement: S4 Fig — (PDF) [file pcbi.1010946.s005.pdf]
