# Supplementary material for: Metheor: Ultrafast DNA methylation heterogeneity calculation from bisulfite read alignments
Source: PLoS Comput Biol. 2023 Mar 20;19(3):e1010946. doi: 10.1371/journal.pcbi.1010946 (PMC10062925; doi:10.1371/journal.pcbi.1010946)
Supplement: S5 Fig — (PDF) [file pcbi.1010946.s006.pdf]

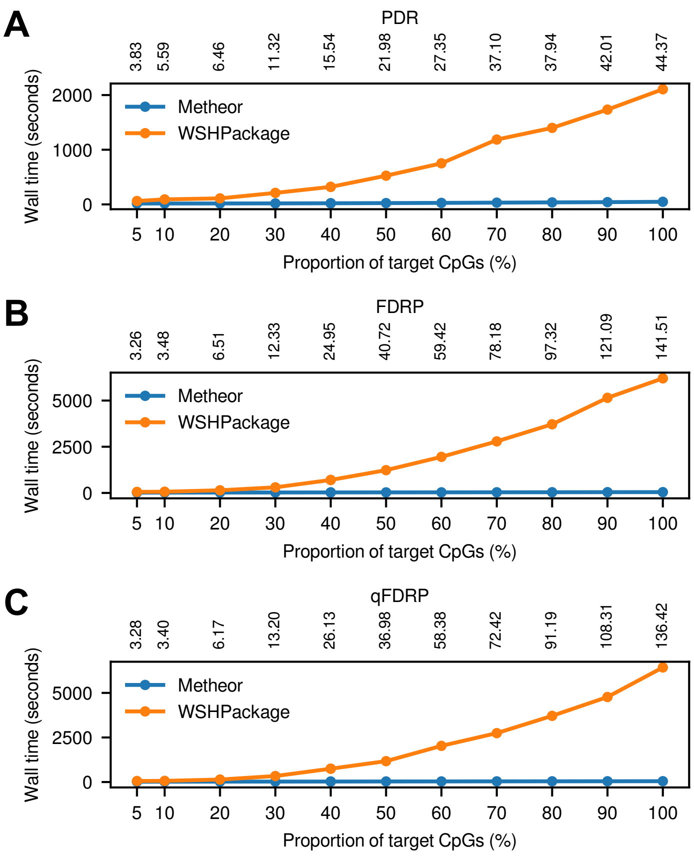

S5 Fig. Benchmarking the running time of Metheor aginst WSHPackage when only a subset of CpGs are considered. Running time of Metheor and WSHPackage for the calculation of (A) PDR, (B) FDRP and (C) qFDRP are computed. Values below the name of each of the measures denote the amount of speedup (in fold) in Metheor compared to WSHPackage.
