# Supplementary material for: Metheor: Ultrafast DNA methylation heterogeneity calculation from bisulfite read alignments
Source: PLoS Comput Biol. 2023 Mar 20;19(3):e1010946. doi: 10.1371/journal.pcbi.1010946 (PMC10062925; doi:10.1371/journal.pcbi.1010946)
Supplement: S6 Fig — (PDF) [file pcbi.1010946.s007.pdf]

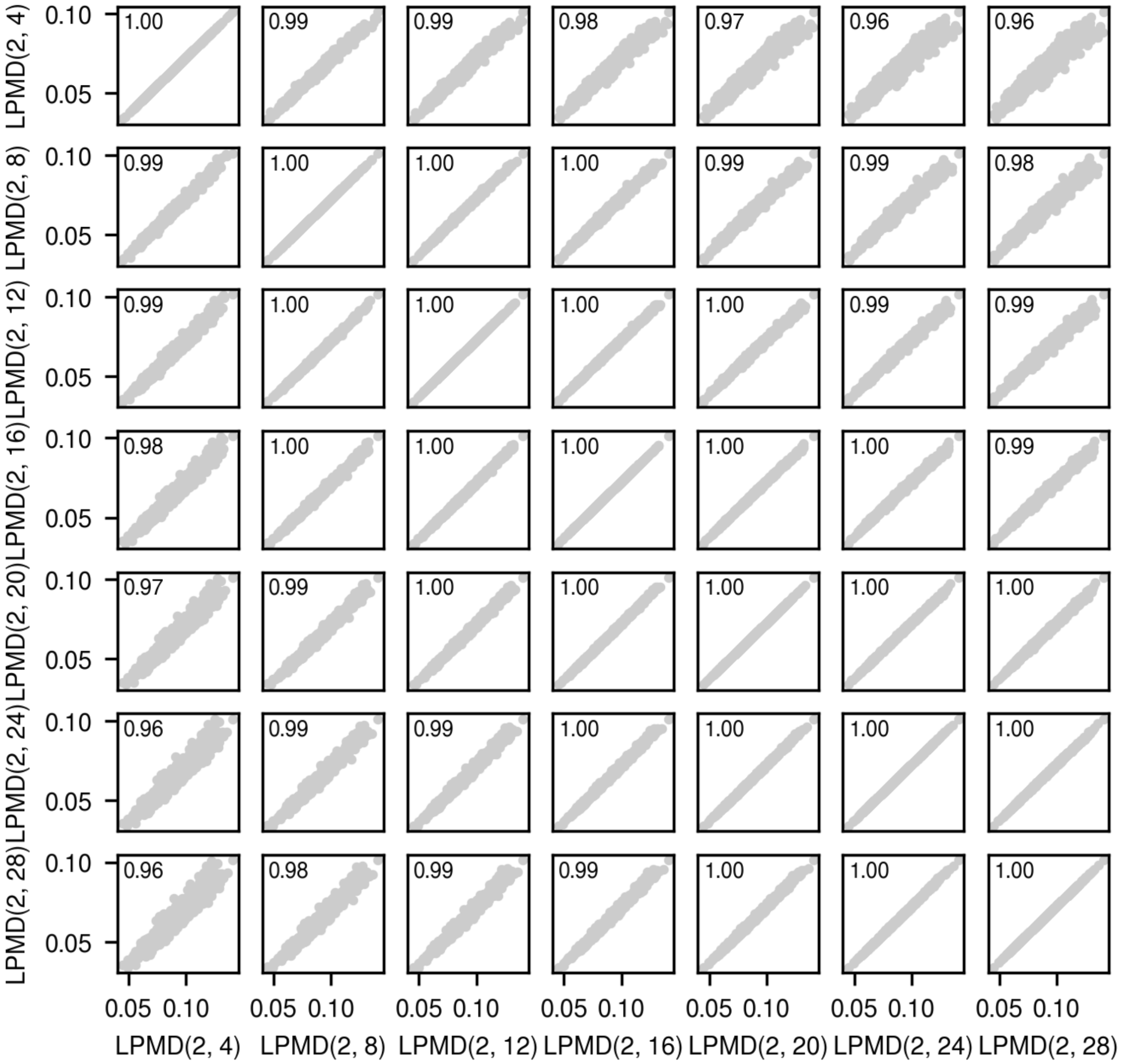

S6 Fig. Robustness of LPMD against the choice of genomic distance window. Each data point represents one of the 928 CCLE cell lines, and the values inside each plot represent Spearman's correlation. Axes labels denote the genomic distance window used for LPMD calculation. For example, LPMD(2, 12) denote that only the CpG pairs that are 2∼12bp away were used to compute LPMD value.
