# Supplementary material for: Metheor: Ultrafast DNA methylation heterogeneity calculation from bisulfite read alignments
Source: PLoS Comput Biol. 2023 Mar 20;19(3):e1010946. doi: 10.1371/journal.pcbi.1010946 (PMC10062925; doi:10.1371/journal.pcbi.1010946)
Supplement: S7 Fig — (PDF) [file pcbi.1010946.s008.pdf]

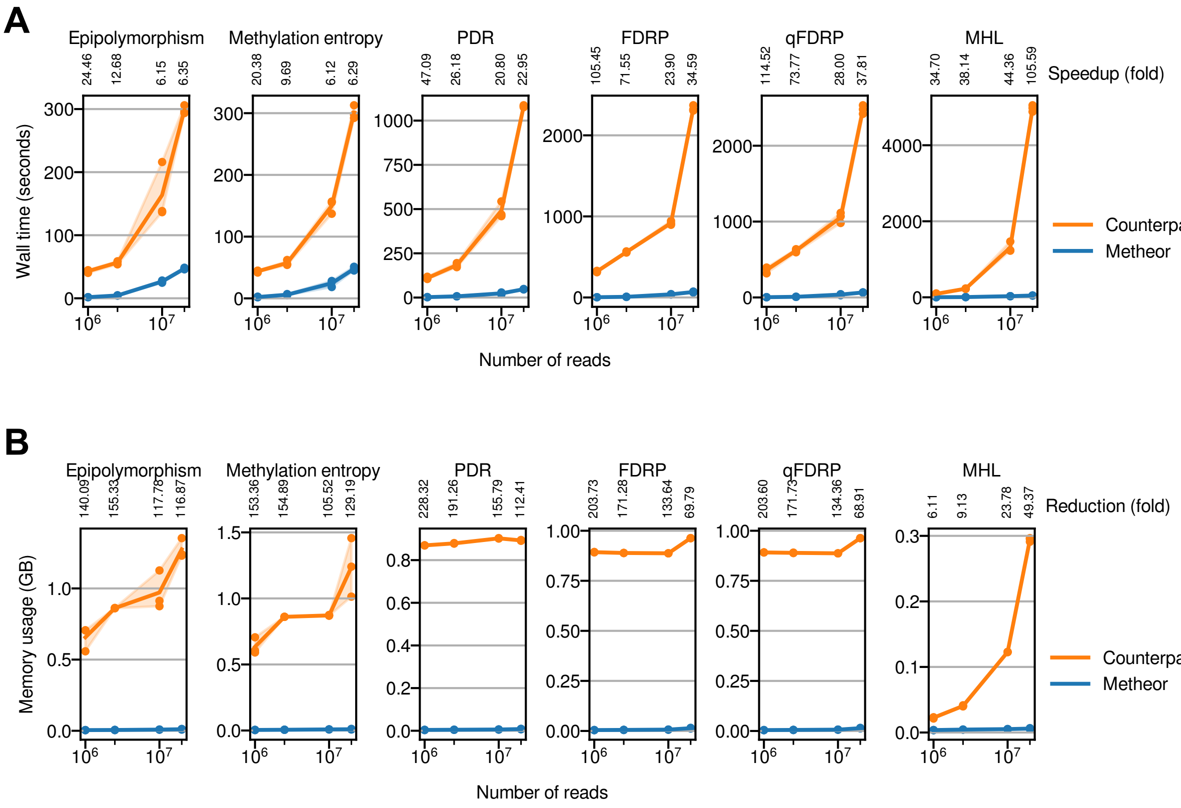

S7 Fig. (A) Benchmarking the running time of Metheor using simulated pseudo-WGBS dataset. Values below the name of each of the measures denote the amount of speedup (in fold) in Metheor compared to its benchmark counterpart. (B) Benchmarking the memory usage of Metheor using simulated pseudo-WGBS dataset. Values below the name of each of the measures denote the amount of memory usage reduction (in fold) in Metheor compared to its benchmark counterpart. All the benchmarking experiments were repeated for three times, except for MHL. Lines denote the average wall time and shades represent the 95% confidence interval. Wall time and memory footprint for MHL computation was measured for only once.
