# Supplementary material for: Metheor: Ultrafast DNA methylation heterogeneity calculation from bisulfite read alignments
Source: PLoS Comput Biol. 2023 Mar 20;19(3):e1010946. doi: 10.1371/journal.pcbi.1010946 (PMC10062925; doi:10.1371/journal.pcbi.1010946)
Supplement: S8 Fig — (PDF) [file pcbi.1010946.s009.pdf]

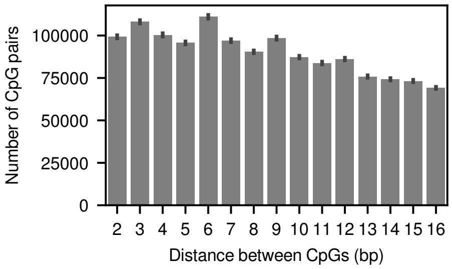

S8 Fig. Distribution of the number of CpG pairs at fixed distances that are used for the computation of genomewide LPMD values of 928 CCLE cell lines. Error bars denote standard errors.
