# Supplementary material for: Metheor: Ultrafast DNA methylation heterogeneity calculation from bisulfite read alignments
Source: PLoS Comput Biol. 2023 Mar 20;19(3):e1010946. doi: 10.1371/journal.pcbi.1010946 (PMC10062925; doi:10.1371/journal.pcbi.1010946)
Supplement: S10 Fig — (PDF) [file pcbi.1010946.s011.pdf]

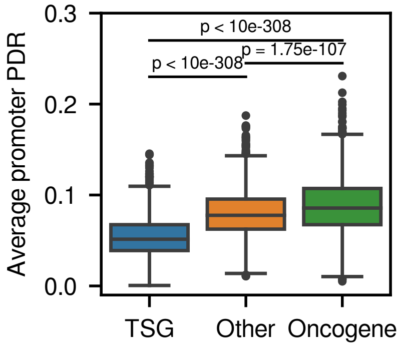

S10 Fig. Promoter PDRs of tumor suppressors and oncogenes. 222 tumor suppressor genes (TSGs) and 206 oncogenes were identified from Cancer Gene Census, and 'Other' denotes genes that are neither TSG nor oncogene. Boxes show the distribution of average promoter PDR values for each cancer cell line. In the boxplot, the center line denotes the median, the upper and lower box limits denote upper and lower quartiles, and the whiskers denote 1.5× interquartile range. P-values are computed using paired t-tests. TSG, tumor suppressor genes.
