# Supplementary material for: Metheor: Ultrafast DNA methylation heterogeneity calculation from bisulfite read alignments
Source: PLoS Comput Biol. 2023 Mar 20;19(3):e1010946. doi: 10.1371/journal.pcbi.1010946 (PMC10062925; doi:10.1371/journal.pcbi.1010946)
Supplement: S9 Fig — (PDF) [file pcbi.1010946.s010.pdf]

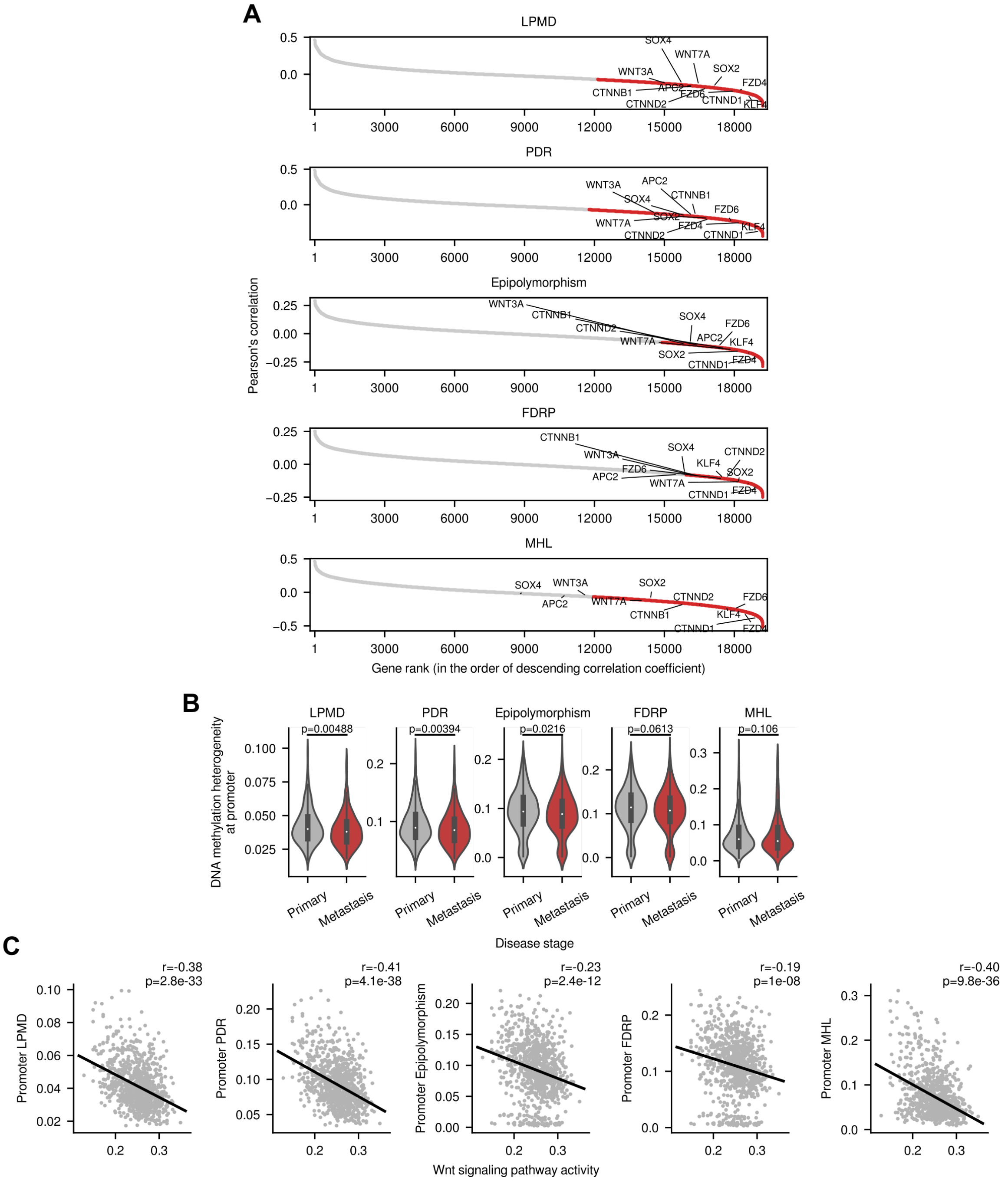

S9 Fig. Association between stemness of cancer cells and other DNA methylation heterogeneity measures. (A) Genes were ranked by the Pearson's correlation between their expression and average methylation entropy levels across promoters. Red dots represent genes having statistically significant correlations (Benjamini-Hochberg adjusted p-value < 0.05). (B) The distribution of promoter DNA methylation heterogeneity levels in primary and metastatic cancer cell lines. P-values from two-tailed independent t-tests are shown. (C) The association between promoter methylation entropy levels and the activity of Wnt signaling pathway. Pearson's correlation coefficients and associated p-values are shown.
