# Supplementary material for: Metheor: Ultrafast DNA methylation heterogeneity calculation from bisulfite read alignments
Source: PLoS Comput Biol. 2023 Mar 20;19(3):e1010946. doi: 10.1371/journal.pcbi.1010946 (PMC10062925; doi:10.1371/journal.pcbi.1010946)
Supplement: Attachment — Submitted filename: response_to_reviewers.docx [file pcbi.1010946.s013.docx]

**Response to reviewer’s comments**

We would like to thank all the reviewers for the insightful and constructive suggestions. According to the comments, we tried to improve the quality of the manuscript through additional methodological descriptions and experimental results. Please see below our detailed response to reviewers’ comments in blue.

**Reviewer #1:**

Major comments

1. The authors point out in the abstract that DNA methylation levels are often overestimated. To tackle this problem, the authors provide a “one-sweep” algorithm (read-centric approach) that reads every sequencing read only one time (line 48). However, it seems that no direct evidence is provided in this manuscript to stress that Metheor can bring a better quantification (or accuracy) of DNA methylation levels by using a read-centric approach than other benchmark algorithms that use the CpG-centric approach. Please clarify this point.

We appreciate the reviewer for pointing out unclear descriptions in the manuscript, which would mislead the readers regarding the motivation and methodology of this study. To convey those ideas in more precise way, we revised the wordings and added some supporting details in the abstract.

In the revised version of the abstract, we wanted to clarify that we were not actually stating DNA methylation levels are overestimated, but we were pointing out that the information contained in phased DNA methylation states (i.e., the ‘pattern’ of DNA methylation states within a single bisulfite sequencing read) have been not fully utilized (or ignored in many cases) in routine DNA methylation analyses, even though such information can help us draw exciting biological findings about “epigenetic heterogeneity” of a cell population. Importantly, the goal of Metheor is to compute the levels of DNA methylation “heterogeneity” in a highly efficient manner, but not to quantify DNA methylation “levels” themselves more accurately. Please note the difference between the “DNA methylation heterogeneity levels” and “DNA methylation levels”, where the former denotes the diversity of DNA methylation states across cells or local heterogeneity of DNA methylation states within a cell, and the latter denotes the percentage of cells having DNA methylation at a specific cytosine. Therefore, the “one-sweep” algorithms suggested in this study are not designed to produce “more accurate” results, but are developed to quantify the levels of DNA methylation heterogeneity in a more efficient manner. Thus, they do not change quantification results at all when compared to benchmark methods **(Figure 2E)**, but reduces the running time effectively **(Figure 2A-D)**.

2. The concept of LPMD is attractive and it is encouraging to see that LPMD is read-length independent. It is however not clear to me, whether a pair of CpGs computed by LPMD are present on the same read or between different reads? In both scenarios, is there any normalization step required for the final readout or any internal control that should be considered? Please clarify this point.

We would like to thank the reviewer for pointing out the unclear methodological description in the manuscript. To clarify that only the pairs of CpG methylation states that are present in the same read are used to compute LPMD, we revised the subsection named “Definition of local pairwise methylation discordance (LPMD)” by adding detailed descriptions **(Line 84~86)**.

Figure R1. Robustness of LPMD against total number of sequencing reads. (A) Schematic illustration of the experimental procedure. Raw sequencing reads were subsampled from Ewing sarcoma RRBS dataset. Five different dataset were generated, each with 0.5M, 2M, 4M, 8M and 15,685,634 (no subsampling) sequencing reads. (B) LPMD values computed from subsampled datasets.

Since LPMD calculation does not depend on read coverage, we thought that LPMD does not require separate normalization or post-processing step. Nevertheless, we agree that it should be tested whether the value of LPMD is robust against the total number of sequencing reads used for the calculation. To this end, we subsampled raw sequencing reads from Ewing sarcoma RRBS dataset used in the study and computed LPMD value for each of the subsampled dataset **(Figure R1A)**. As a result, we observed that even only with 3.1% (500,000 out of 15,685,634) of raw sequencing reads the LPMD value did not deviate a lot from the LPMD value computed from full dataset (~0.085) **(Figure R1B)**. Therefore, we could show that LPMD is robust against the total number of sequencing reads.

3. Please include more information about the choice of the distance of CpG pairs used in LPMD (line 104) and the influence on LPMD values. In other words, can the authors provide results by simulating different CpG pair distances?

We would like to thank the reviewer for the suggestion. In this study, the 2~16bp genomic distance window was heuristically chosen for LPMD calculation since we initially thought that only by focusing on sufficiently narrow genomic window, shorter than 16bp for example, we can effectively capture the differential levels of local homogeneity of DNA methylation states among the cancer cell lines. Moreover, the length of sequencing reads for CCLE RRBS dataset was 29nt. Hence, methylation states of CpG pairs farther than 29bp away cannot be captured simultaneously by a single sequencing read. These reasons made us use CpG pairs within 16bp genomic windows.

To examine the effect of the genomic window size used in LPMD calculation, we show that the order LPMD values among 928 cancer cell lines are highly robust to the choice of genomic distance window (up to 28bp, **Figure R2**), suggesting that the choice of distance window is not critical for LPMD analyses as long as the same window is used for all samples subjected to the comparison of LPMD values. We revised subsection “Definition of local pairwise methylation discordance (LPMD)” in the manuscript, and added subsection “1.9 Robustness of LPMD against the choice of genomic distance window” **(Line 109~111)** and **Supplementary Figure 6** to the Supplementary Material.

Figure R2. Robustness of LPMD against the choice of genomic distance window. Each data point represents one of the 928 CCLE cell lines, and the values inside each plot represent Spearman’s correlation. Axes labels denote the genomic distance window used for LPMD calculation. For example, LPMD(2, 12) denote that only the CpG pairs that are 2~12bp away were used to compute LPMD value.

4. Please provide more detail about how to compute genomewide methylation levels (line 205) shown in Figure 3. This information could be helpful to understand why LPMD gives different profiles of methylation levels.

We appreciate this comment for pointing out the incomplete description about genomewide methylation levels. We agree that the manuscript needs more detailed description for method to compute genomewide methylation levels. To this end, we added a brief description after the term “genomewide methylation levels” **(Line 230)** in subsection “Characteristics of the local DNA methylation disorder captured by LPMD across 928 cancer cell lines.”

Again, we also note that LPMD represents the extent of DNA methylation heterogeneity, but not the level of DNA methylation itself (Please refer to our response for comment #1 of reviewer #1).

5. The authors point out the inconsistent patterns between genomewide methylation levels and the LPMD level (Fig 3B); however, the interpretation does not appear to be sound (lines 212-218). In addition, the scale of the methylation level computed by LPMD is relatively low compared to the genomewide methylation level. It may lead to different interpretation of results. The authors should stress this point.

We would like to thank the reviewer for this valuable comment. First of all, it is important to note that “genomewide methylation level” and “LPMD” does not quantify the same biological phenomenon, so directly comparing the absolute levels of those two measures may not be reasonable. Genomewide methylation level is computed as an average DNA methylation level across all CpGs throughout the genome, while LPMD quantifies the probability of two CpG sites at specific genomic distance having different DNA methylation states. Due to the well-known local homogeneity of DNA methylation states (Eckhardt et al., 2006, Saito et al., 2015) (i.e., two nearby CpGs have identical methylation states in general), the absolute level of LPMD is usually lower than ~0.1 as you can see in **Figure 3A and 3G-I**. In other words, the probability that two CpG sites within 16bp from each other having different methylation states is lower than ~10%. On the other hand, the distribution of methylation levels throughout the genome is usually bimodal around ~0.1 and ~0.9, thus their average values reside within 0.4~0.6 as shown in **Figure 3A**. We revised subsection “Characteristics of the local DNA methylation disorder captured by LPMD across 928 cancer cell lines” in the manuscript to deliver the discussion above more clearly.

6. The authors use the gene dnmt3a to illustrate the correlations between gene expression and methylation levels computed by LPMD and another method, respectively (Fig 3C-3F). However, the authors do not discuss about the possible reasons caused this discrepancy. Is it biological-relevant or due to different methods used to calculate methylation levels?

We thank the reviewer for suggesting further biological discussion regarding the correlation between the expression of *DNMT3A* and genomewide LPMD or genomewide methylation levels. Although it needs clearer experimental support, our conjecture is that the prominent negative correlation between the expression level of *DNMT3A* and LPMD is due to the unique enzymatic property of DNMT3A that is dependent on the oligomeric state of the protein (Holz-Schietinger et al., 2011). More specifically, homotetrameric DNMT3A shows “processive” *de novo* methylation, where the methylation is added to adjacent CpGs consecutively. On the other hand, homodimeric DNMT3A shows “distributive” catalysis, where the enzyme complex frequently dissociates from the DNA after catalysis. As the oligomeric state of homomer complex is known to heavily dependent on monomer concentration (Bergendahl et al., 2017), we speculate that the increase of *DNMT3A* gene expression will make intracellular DNMT3A oligomeric state biased toward homotetramers, thus increasing the local homogeneity of DNA methylation states. As suggested by the reviewer, we added this discussion to subsection “Characteristics of the local DNA methylation disorder captured by LPMD across 928 cancer cell lines” **(Line 268~276)** in the manuscript.

7. In Figure 3G and 3H, the increasing magnitude of average LPMD values shrinks as the distance is greater than 4 bp, which could imply that a relatively lower frequency of CpG pairs can be found beyond this distance or there are missing pairs of CpGs. Are the authors aware of the coverage of counted methylated CpGs using LPMD?

We thank the reviewer for the suggestion. We agree that if the coverage of CpG pairs at a fixed distance is not sufficiently high, the LPMD value for that distance would not be reliable. To examine whether there were sufficient number of CpG pairs at 2~16bp away to compute LPMD values, we counted the number of CpG pairs and confirmed that we had sufficient amount of them (>69,000 pairs for every distance in 2~16bp). We added a figure (**Supplementary Figure 8**) showing the number of CpG pairs at fixed distances.

8. It is not clear to me, as the authors compute the discordance of methylation states with increasing distance between CpG pairs (Fig 3G & 3H), do the authors take all pairs of methylated CpGs called within the indicated distance or only CpG pairs with indicated distance? If it is the first case, it should not be surprising that probabilistically the longer the distance between CpG pairs is, the higher the LPMD level is when we choose a window between 2 - 16 bp (considering that the average length of CpG is between 300 - 3000 bp). The authors please clarify this point.

We are grateful for this comment as we can further clarify the rationale behind **Figure 3G & 3H.** In those figures, we consider all pairs of CpGs that are exactly placed at specified genomic distance. For example, when we compute LPMD for distance 2bp, we consider every pairs of CpGs that are exactly 2bp away from each other. If the methylation states of two CpGs are independent, the probability of the two CpGs having different methylation states will be independent of the genomic distance between CpGs. In this context, we concluded that **Figure 3G & 3H** reflects the tendency towards co-methylation of CpG placed at short distance.

9. All correlation results (Fig 3E & 3F; Fig 4D & 4E) seem to be quite noisy. Without regression lines, I barely consider there is any correlation present. At this stage, it is not clear whether this phenomenon is due to LPMD or the variation of gene expression from different genes or tissue samples, or other reasons. Therefore, I suggest that the authors turn some down of their claims stated in the hypotheses proposed in line 243 and line 266.

Thank you for your suggestion. We agree that the magnitude of the correlation coefficient is not strikingly high, so we toned down the argument regarding correlation coefficients in subsection “Characteristics of the local DNA methylation disorder captured by LPMD across 928 cancer cell lines” in the manuscript.

10. The accuracy of methylation calls is crucial to unveil the correct sites of methylation occurred. It is not clear whether the algorithms (provided in Supplementary information) used in Metheor contain the parameters to minimize the probability of false positives. If so, please highlight the command lines in the algorithms. If not, please explain how Metheor counteracts the issue of methylation miscalls.

We thank the reviewer for this intriguing comment. We indeed agree that erroneous DNA methylation calls will hamper accurate computation of DNA methylation heterogeneity levels. In most bisulfite sequencing analyses, DNA methylation states (i.e., methylated or unmethylated) of CpG dinucleotides are determined using bisulfite-converted reads aligned to bisulfite-converted reference genome. In this procedure, there are two sources of DNA methylation call errors: incomplete bisulfite conversion and sequencing base-call errors. Since incomplete bisulfite conversion does not convert unmethylated cytosine (C) to uracil (U) (and eventually to thymine (T)), it will result in false-positive DNA methylation calls. On the other hand, sequencing base-call errors can lead to both false-positive and false-negative calls. However, in general, both bisulfite conversion error rate and sequencing base-call error rates are reasonably low (<0.5% and ~0.1%, respectively), so routine bisulfite sequencing analyses usually do not attempt to correct for methylation miscalls. Moreover, to show that Metheor produces exactly identical results compared to reference implementations for epipolymorphism, methylation entropy, PDR, FDRP, qFDRP and MHL, we did not add functionality that corrects methylation miscalls.

11. Please clarify how concordant CpG pairs are sampled that stated in line 2 in Algorithm 3 (Supplementary results).

We would like to thank the reviewer for pointing out an unclear description in the algorithm. We added some detailed comments in Algorithms 3 **(Line 3)** and 4 **(Line 21 and 24)** to clarify how we compute the number of concordant/discordant CpG pairs from the bisulfite read alignment result. Please note that while computing LPMD, we do not sample CpG pairs or sequencing reads, but just use the entire aligned reads **(Line 5 in Algorithm 3)** as well as every CpG pair in a single sequencing read **(Line 6 in Algorithm 4)**.

Minor comment

1. I suggest the authors move the section “Definition of local pairwise methylation discordance (LPMD), line 96” in front of the section “Simulation of sequencing data with different read lengths, line 79”.

We agree that moving the subsection “Definition of local pairwise methylation discordance (LPMD)” forward will emphasize the importance of the new DNA methylation heterogeneity measure better. We revised the structure of “Methods” section accordingly.

**Reviewer #2:**

Major comments

1. Cancer cells usually have global low DNA methylation and local high DNA methylation, especially on cancer repressors. Can the authors illustrate the relationships between cancer repressors and DNA methylation heterogeneity?

Figure R3. Promoter PDRs of tumor suppressors and oncogenes. Boxes show the distribution of average promoter PDR values for each cancer cell line. In the boxplot, the center line denotes the median, the upper and lower box limits denote upper and lower quartiles, and the whiskers denote 1.5× interquartile range. P-values are computed using paired t-tests. ‘Other’ denotes genes that are neither TSG nor oncogene. TSG, tumor suppressor genes.

We would like to appreciate the reviewer for this insightful and exciting comment. Given that level of promoter DNA methylation negatively correlates with the expression of corresponding genes, it would be particularly interesting to examine the DNA methylation heterogeneity levels at the promoters of tumor suppressor genes in cancer cell lines. To this end, we first identified 222 tumor suppressor genes (TSGs) and 206 oncogenes from Cancer Gene Census. Then, for each of the 928 cancer cell lines, average promoter PDR values for separate gene groups (TSGs, oncogenes and other genes) were computed. Strikingly, we found that PDRs at the promoters of TSGs are significantly lower than that of oncogenes and the rest of the genes (**Figure R3**). As the reviewer pointed out, promoters of tumor suppressor genes are usually hypermethylated in cancer cells. Given that the local disorder of promoter DNA methylation states (measured by PDR) of TSGs are kept remarkably low in cancer cell lines, we can hypothesize that it is important for cancer cell lines to faithfully transmit the hypermethylated DNA methylation states to daughter cells. In stark contrast, promoter PDR of oncogenes are significantly higher than that of TSGs, suggesting that more variability is allowed for the epigenetic regulation of oncogenes. We think that this finding is a good starting point to understand the different role of epigenetic regulation of TSGs and oncogenes in the context of cancer evolution.

To facilitate further studies originating from the discovery, we added this result to “Discussion” section **(Line 310~313)** in the manuscript and **Supplementary Figure 10**.

2. Can the authors get similar results based on the 928 cancer cell lines if they use different DNA methylation heterogeneity measures?

Figure R4. Association between stemness of cancer cells and other DNA methylation heterogeneity measures. (A) Genes were ranked by the Pearson’s correlation between their expression and average methylation entropy levels across promoters. Red dots represent genes having statistically significant correlations (Benjamini-Hochberg adjusted p-value < 0.05). (B) The distribution of promoter DNA methylation heterogeneity levels in primary and metastatic cancer cell lines. P-values from two-tailed independent t-tests are shown. (C) The association between promoter methylation entropy levels and the activity of Wnt signaling pathway. Pearson’s correlation coefficients and associated p-values are shown.

Thank you for the suggestion. We agree that it would be interesting to examine whether we can get similar results using different DNA methylation heterogeneity measures. To this end, we reproduced the experiments conducted in subsection “Homogeneity of DNA methylation patterns across cell population is associated with the stemness of cancer cells” using five other measures, namely LPMD, PDR, epipolymorphism, FDRP and MHL. As a result, we observed the homogeneity of DNA methylation states, captured by those additional measures, was also associated with the expression of stemness-related genes **(Figure R4A)**, and metastatic cell lines exhibited lower DNA methylation heterogeneity in terms of LPMD, PDR and epipolymorphism **(Figure R4B)**. Finally, we observed that Wnt pathway activity levels were negatively correlated with DNA methylation heterogeneity as in the case of methylation entropy **(Figure R4C)**. Notably, LPMD, PDR and MHL even showed clearer tendency of negative correlation than methylation entropy. Altogether, we could conclude that cancer cell lines with increased stemness and metastatic cell lines display decreased DNA methylation heterogeneity in terms of both within-cell and cell-to-cell heterogeneity. We added these results to **Supplementary Figure 9** and added relevant discussions to subsection “Homogeneity of DNA methylation patterns across cell population is associated with the stemness of cancer cells”.

Minor comments

1. The authors should make the figures clearer.

Thank you for the comment. According to the suggestion, we increased the resolution of the figures.

**References**

Bergendahl, L. T., & Marsh, J. A. (2017). Functional determinants of protein assembly into homomeric complexes. Scientific reports, 7(1), 4932.

Eckhardt, F., Lewin, J., Cortese, R., Rakyan, V. K., Attwood, J., Burger, M., ... & Beck, S. (2006). DNA methylation profiling of human chromosomes 6, 20 and 22. Nature genetics, 38(12), 1378-1385.

Holz-Schietinger, C., Matje, D. M., Harrison, M. F., & Reich, N. O. (2011). Oligomerization of DNMT3A controls the mechanism of de novo DNA methylation. Journal of Biological Chemistry, 286(48), 41479-41488.

Saito, D., & Suyama, M. (2015). Linkage disequilibrium analysis of allelic heterogeneity in DNA methylation. Epigenetics, 10(12), 1093-1098.
