# Supplementary material for: Metheor: Ultrafast DNA methylation heterogeneity calculation from bisulfite read alignments
Source: PLoS Comput Biol. 2023 Mar 20;19(3):e1010946. doi: 10.1371/journal.pcbi.1010946 (PMC10062925; doi:10.1371/journal.pcbi.1010946)
Supplement: Attachment — Submitted filename: [reviewer comments]-PLOS Computational Biology.pdf [file pcbi.1010946.s012.pdf]

Comments for the Authors

PCOMPBIOL-D-22-01380

Metheor: Ultrafast DNA methylation heterogeneity calculation from bisulfite read alignments

Authors: Dohoon Lee, Bonil Koo, Jeewon Yang, Sun Kim

The authors present a new bioinformatic toolkit, Metheor, aiming to compute average DNA methylation levels in a quantitative manner and visualize the heterogeneity of DNA methylation profiles at a genomewide scale.

In this manuscript, the authors clearly explain the rationale of Metheor and benchmark this method by comparing their analytical outputs to which resulted from WSHPackage (Table 1). The authors provide evidence showing that Metheor requires a short operation time and low memory usage to analyze bisulfite read alignments (Figure 2). It is essential to stress that the main methodological difference between Metheor and other existing software is that Metheor allows to additionally look for the discrepancy of methylation states between two CpGs (so-called local pairwise methylation discordance, LPMD). Finally, the authors attempt to bring more biological impacts on the heterogeneity of DNA methylation across 928 cell lines using Metheor. The author test both intracellular- (Figure 3) and intercellular DNA methylation discordance (Figure 4) represented by outputs computed from LPMD and methylation entropy, respectively, and propose their theoretical hypotheses on cancer biology and DNA methylation.

Overall, this manuscript is well-written and brings the community a new toolkit for analyzing bisulfite read alignment readouts. There are a few issues that the authors may try to clarify, as suggested below:

Major comments:

1. The authors point out in the abstract that DNA methylation levels are often overestimated. To tackle this problem, the authors provide a "one-sweep" algorithm (read-centric approach) that reads every sequencing read only one time (line 48). However, it seems that no direct evidence is provided in this manuscript to stress that Metheor can bring a better quantification (or accuracy) of DNA methylation levels by using a read-centric approach than other benchmark algorithms that use the CpG-centric approach. Please clarify this point.

2. The concept of LPMD is attractive and it is encouraging to see that LPMD is read-length independent. It is however not clear to me, wether a pair of CpGs computed by LPMD are present on the same read or between different reads? In both scenarios, is there any normalization step required for the final readout or any internal control that should be considered? Please clarify this point.

3. Please include more information about the choice of the distance of CpG pairs used in LPMD (line 104) and the influence on LPMD values. In other words, can the authors provide results by simulating different CpG pair distances?

4. Please provide more detail about how to compute genomewide methylation levels (line 205)shown in Figure 3. This information could be helpful to understand why LPMD gives different profiles of methylation levels.

5. The authors point out the inconsistent patterns between genomewide methylation levels and the LPMD level (Fig 3B); however, the interpretation does not appear to be sound (lines 212-218). In addition, the scale of the methylation level computed by LPMD is relatively low compared to the genomewide methylation level. It may lead to different interpretation of results. The authors should stress this point.

6. The authors use the gene *dnmt3a* to illustrate the correlations between gene expression and methylation levels computed by LPMD and another method, respectively (Fig 3C-3F). However, The authors do not discuss about the possible reasons caused this discrepancy. Is it biological-relevant or due to different methods used to calculate methylation levels?

7. In Figure 3G and 3H, the increasing magnitude of average LPMD values shrinks as the distance is greater than 4 bp, which could imply that a relatively lower frequency of CpG pairs can be found beyond this distance or there are missing paris of CpGs. Are the authors aware of the coverage of counted methylated CpGs using LPMD?

8. It is not clear to me, as the authors compute the discordance of methylation states with increasing distance between CpG pairs (Fig 3G & 3H), do the authors take all pairs of methylated CpGs called within the indicated distance or only CpG pairs with indicated distance? If it is the first case, it should not be surprising that probabilistically the longer the distance between CpG pairs is, the higher the LPMD level is when we choose a window between 2 - 16 bp (considering that the average length of CpG is between 300 - 3000 bp). The authors please clarify this point.

9. All correlation results (Fig 3E & 3F; Fig 4D & 4E)seem to be quite noisy. Without regression lines, I barely consider there is any correlation present. At this stage, it is not clear whether this phenomenon is due to LPMD or the variation of gene expression from different genes or tissue samples, or other reasons. Therefore, I suggest that the authors turn some down of their claims stated in the hypotheses proposed in line 243 and line 266.

10. The accuracy of methylation calls is crucial to unveil the correct sites of methylation occurred. It is not clear whether the algorithms (provided in Supplementary information) used in Metheor contain the

parameters to minimize the probability of false positives. If so, please highlight the command lines in the algorithms. If not, please explain how Metheor counteracts the issue of methylation miscalls.

11. Please clarify how concordant CpG pairs are sampled that stated in line 2 in Algorithm 3 (Supplementary results).

Minor comment:

1. I suggest the authors move the section “Definition of local pairwise methylation discordance (LPMD), line 96” in front of the section “Simulation of sequencing data with different read lengths, line 79”.
